# Time to blood culture positivity as a predictor of clinical outcomes and severity in adults with bacteremic pneumococcal pneumonia

**DOI:** 10.1371/journal.pone.0182436

**Published:** 2017-08-07

**Authors:** Catia Cillóniz, Adrian Ceccato, Cristina de la Calle, Albert Gabarrús, Carolina Garcia-Vidal, Manel Almela, Alex Soriano, José Antonio Martinez, Francesc Marco, Jordi Vila, Antoni Torres

**Affiliations:** 1 Department of Pneumology, Institut Clinic del Tórax, Hospital Clinic of Barcelona—Institut d'Investigacions Biomèdiques August Pi i Sunyer (IDIBAPS), University of Barcelona, Ciber de Enfermedades Respiratorias (CIBERES), Barcelona, Spain; 2 Department of Pneumology, National Hospital Alejandro Posadas, Palomar, Argentina; 3 Department of Infectious Disease, Hospital Clinic of Barcelona, Barcelona, Spain; 4 Department of Microbiology, Hospital Clinic of Barcelona, Barcelona, Spain; University of Western Australia, AUSTRALIA

## Abstract

**Objectives:**

We aimed to investigate the association between the time to positivity of blood culture (TTP) with clinical outcome and severity of pneumococcal bacteremic pneumonia.

**Methods:**

Prospective observational study carried out in 278 hospitalized adult CAP patients with positive blood culture for *Streptococcus pneumonia* (2003–2015).

**Results:**

A total of 278 cases of bacteremic pneumococcal pneumonia were analyzed, median age 62 (46; 79) years. Fifty-one percent of the cases had PSI IV-V. Twenty-one (8%) died within 30-days after admission. The analysis of the TTP showed that the first quartile of the TTP (9.2h) was the best cut-off for differentiating 2 groups of patients at risk, early (TTP <9.2 h) and late (TTP ≥9.2 h) detection groups (AUC 0.66 [95% CI 0.53 to 0.79]). Early TTP was associated with a statistically significant risk of invasive mechanical ventilation (18% vs. 6%, p = 0.007), longer length of hospital stay (12 days vs. 8 days, p<0.001), higher in-hospital mortality (15% vs. 4%, p = 0.010), and 30-day mortality (15% vs. 5%, p = 0.018). After adjustment for potential confounders, regression analyses revealed early TTP as independently associated with high risk of invasive mechanical ventilation (OR 4.60, 95% CI 1.63 to 13.03), longer length of hospital stay (β 5.20, 95% CI 1.81 to 8.52), higher in-hospital mortality (OR 5.35, 95% CI 1.55 to 18.53), and a trend to higher 30-day mortality (OR 2.47, 95% CI 0.85 to 7.21) to be a contributing factor.

**Conclusion:**

Our results demonstrate that TTP is an easy to obtain surrogate marker of the severity of pneumococcal pneumonia and a good predictor of its outcome.

## Introduction

*Streptococcus pneumoniae* remains the most frequent cause of community-acquired pneumonia (CAP) [1,2]. Bacteremia is documented in 25% of cases [3] and their mortality is 15% to 26% greater than in non-bacteremic patients [4]. The identification of early predictors of worse outcome in patients with bacteremic CAP due to *S*. *pneumoniae* is therefore of utmost importance. There is evidence about the association between the high bacterial load and worse clinical outcomes in invasive pneumococcal pneumonia [5,6]. This evidence suggests that determination of pneumococcal load has a clinical utility. Some previous studies suggest that time to positivity (TTP) of blood culture may provide early clues about microorganisms involved and the source of bacteremia [7]. Also, TTP is inversely associated with blood bacterial load (8) and is therefore a reasonable marker of more severe disease and a potential early predictor of mortality.

Two previous studies analyzed the TTP in children and adults with *S*. *pneumoniae* bacteremia [8,9]. The experience in children included 175 episodes from different sources (meningitis, pneumonia) and no association was found between TTP and clinical or laboratory parameters except that of the 150 patients seen in the emergency department, those in the first decile were significantly more likely to be admitted to the hospital than were patients in the 10th decile (10 of 15 patients [67%] vs. 5 of 16 patients [31%]; p = 0.02) [9]. In contrast, the experience in adults included 105 episodes and shorter TTP was associated with immunosuppression, severe sepsis and shock, meningitis or ICU admission [8]. However, the number of patients with CAP included in these studies was low and the authors did not evaluate the potential relationship with mortality.

*S*. *pneumoniae* is capable of producing a different phenotypic expression, depending on the capsular serotype; it is known that certain serotypes can cause a more invasive disease than others [3]. The higher virulence of these serotypes could be associated with a shorter TTP.

The aim of our study was to evaluate the TTP in prospectively collected episodes of bacteremic CAP due to *S*. *pneumoniae* from 2003 to 2015 and to correlate the TTP with the severity of CAP, the *S*. *pneumoniae* serotype and the length of hospital stay, in-hospital mortality rate, 30-day mortality rate, ICU admission rate, ICU mortality rate, length of stay in ICU, and need of mechanical ventilation.

## Materials and methods

### Ethics statement

The study was approved by the Ethics Committee of Hospital Clinic of Barcelona, Spain (Register: 2009/5451). Written informed consent was waived due to the non-interventional design. Patients’ identification remained anonymous.

### Study design and patients

We performed a prospective observational study including all adults consecutively admitted between 2003 to 2015 with a diagnosis of community-acquired pneumococcal pneumonia to the Hospital Clinic of Barcelona, Spain, an 800-bed third-level hospital covering an urban population of 540,000 inhabitants. We excluded patients who were immunosuppressed, receiving immunosuppressant (those taking >10 mg/day of prednisone or cytotoxic therapy) and all patients known to have human immunodeficiency virus infection.

### Data collection and evaluation

At the initial visit, patients underwent a complete clinical history and physical examination. Patients were stratified into risk classes using the validated prediction rule calculated according to the Pneumonia Severity Index (PSI) score [10]. We also calculated the CURB-65 [11] and the sequential organ failure assessment (SOFA) [12] scores at admission. Empirical antibiotic treatment was administered according to the Infectious Disease Society of America/American Thoracic Society (IDSA/ATS) guidelines for management of CAP [13]. All surviving patients were visited at 30–40 days after discharge.

### Microbiological evaluation and diagnostic criteria

Regular sampling included sputum specimens, two blood cultures, urine samples for detection of *S*. *pneumoniae* (BinaxNOW *S*. *pneumoniae* Urinary Antigen Test; Emergo Europe, The Hague, The Netherlands) and *Legionella pneumophila* serogroup 1 (BinaxNOW *L*. *pneumophila* Urinary Antigen Test; Trinity Biotech, Bray, Ireland). Samples from pleural fluid puncture, tracheobronchial aspiration (TBAS) and blind bronchoalveolar lavage (BAL) were obtained according to the judgment of the attending physician.

The etiology of pneumococcal pneumonia was determined in cases with a positive valid sputum culture, positive blood culture; positive pleural fluid and transthoracic needle aspiration cultures; positive urinary antigen for *S*. *pneumoniae*; bacterial growth in cultures of TBAS ≥10^5^ CFU/ml, in Protective Brush Sample (PBS) ≥10^3^ CFU/ml, and in BAL ≥10^4^ CFU/ml. For the purpose of this study we only included patients with positive blood culture.

Blood cultures were processed by the BACTEC 9240 system (Becton-Dickinson, MD, USA), and vials were loaded into the machine around the clock. Volumes between 8 to 10 ml of blood samples were inoculated into aerobic and anaerobic vials. The vials used were the resin-containing BACTEC Plus Aerobic/F and BACTEC Plus Anaerobic/F or the non-resin-containing BACTEC Standard 10 Aerobic/F and BACTEC Lytic/10 Anaerobic/F. The incubation period was 5 days before being discarded as negative. The identification of microorganisms isolated from positive cultures was performed according to conventional methods.

Strains were initially screened for antimicrobial susceptibility using Sensititre (Trek Diagnostic Systems Ltd, West Sussex, England). Penicillin and other antibiotic susceptibilities were defined according to EUCAST criteria [14]. For *S*. *pneumoniae* isolates, minimum inhibitory concentrations (MICs) were determined using the Sensititre for penicillin, cefotaxime, ceftriaxone, cefepime, imipenem, meropenem, erythromycin, clindamycin, levofloxacin, and vancomycin. Results were interpreted according to EUCAST criteria [14].

Pneumococcal isolates were identified by standard microbiological methods. All strains isolated from normally sterile sites were routinely frozen at -70°C in skimmed milk until serotype detection was performed. Isolates were serotyped at the Spanish Reference Laboratory for Pneumococci (Majadahonda, Madrid, Spain) by using the Quellung reaction (antisera provided by the Statens Serum Institute Copenhagen, Denmark) and/or dot blot analysis [15].

### Definitions

Pneumonia was defined as the presence of a new infiltrate on a chest radiograph together with clinical symptoms that were suggestive of lower respiratory tract infection (e.g., fever, cough, sputum production, pleuritic chest pain).

Prior antibiotic treatment was considered when antibiotics had been taken in the previous month.

Bacteremic pneumococcal pneumonia was defined as the presence of a positive blood culture for *S*. *pneumoniae*.

The TTP was determined from the time interval between the start of incubation and the detection of microbial growth in peripheral blood, as documented using an automated monitoring system. When multiple cultures were positive only the shortest TTP was selected for analysis.

Pneumococcal serotypes were grouped according to the invasive potential: low (serotypes 3, 6A, 6B, 19A, 19F AND 23F), medium (4, 9N, 9V, 14 and 18C) and high (1, 5 and 7F) [16–18].

Severe CAP was defined according to the IDSA/ATS guidelines criteria [13].

Pulmonary complications were defined by the presence of pleural effusion, empyema, or multilobar infiltrates. Extra-pulmonary complication was defined by the presence of septic shock and acute renal failure.

### Clinical outcomes

The primary outcome was in-hospital mortality. Secondary outcomes included length of hospital stay, 30-day mortality, ICU admission, length of stay in ICU, ICU mortality, and need of mechanical ventilation.

### Statistical analysis

Data are shown as number and percentage of patients for categorical variables and median (Quartile 1 [Q_1_]; Quartile 3 [Q_3_]) for continuous variables with non-normal distribution or mean (standard deviation [SD]) for those with normal distribution. Categorical variables were compared using the X^2^ test or the Fisher exact test. Continuous variables were compared using the t-test or the nonparametric Mann-Whitney test. A receiver operating characteristic (ROC) curve was constructed to determine the best cut-point for TTP to predict in-hospital mortality. Youden’s index [19] was defined for all points along the ROC curve, and the maximum value of the index was used as a criterion for selecting the optimum cut-off point. Regression analyses [20,21] were used to examine the associations between outcomes (a linear regression analysis for length of hospital stay, two logistic regression analyses for in-hospital mortality and 30-day mortality, and a multinomial logistic regression analysis for non-invasive or invasive mechanical ventilation) and risk factors. In a first step, each risk factor was tested individually. In a second step, all risk factors which showed an association in the univariate model (p<0.10) were added into the multivariate model. Finally, a backward stepwise selection (p_in_<0.05, p_out_<0.10) was used to determine factors associated with outcome. The beta coefficient (β) and 95% confidence interval (CI) and the odds ratio (OR) and 95% (CI) were calculated where applicable. The Hosmer-Lemeshow goodness-of-fit test was performed to assess the overall fit of the logistic regression models, the R^2^ for the linear regression model, and the Cox and Snell R^2^ and the Nagelkerke R^2^ for the multinomial logistic regression model. Internal validation of the prediction models was conducted using ordinary nonparametric bootstrapping with 1,000 bootstrap samples and bias-corrected, accelerated 95% CIs [22]. The area under the ROC curve of the multivariate models to predict in-hospital mortality, 30-day mortality, non-invasive and invasive mechanical ventilation were calculated. Simple imputations of random effects were used, if necessary, for variables with missing values. The level of significance was set at 0.05 (2-tailed). All analyses were performed using IBM SPSS Statistics version 22.0 (Armonk, New York, USA).

## Results

### Participants

**Fig 1:**
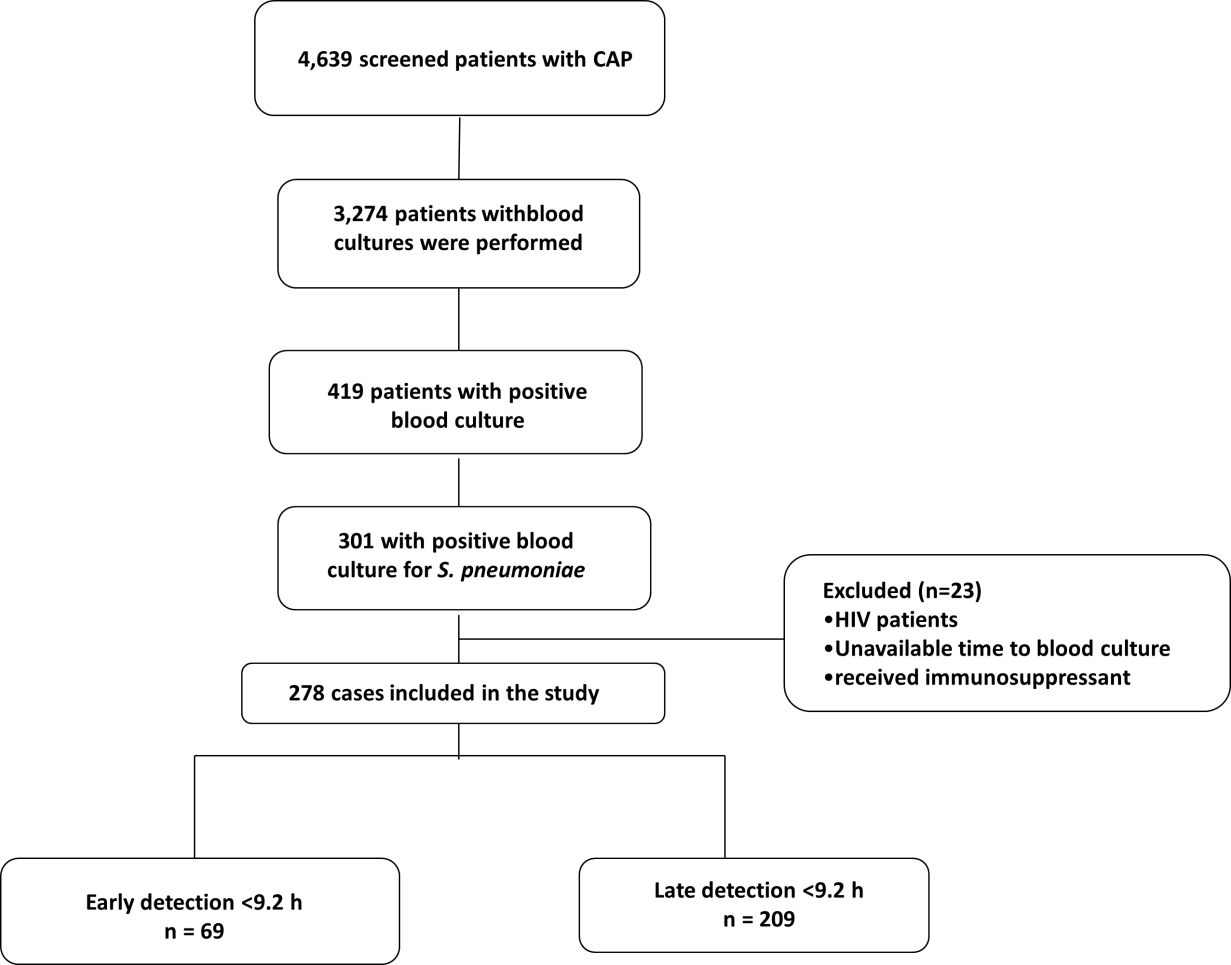
Flow diagram of the selected population.

During the study period, 4,639 patients were admitted in the Emergency Department with the diagnosis of CAP. Blood cultures were performed on 3,274 (71%) and were positive in 419 (13%). Of these, 301 (72%) were positive for *S*. *pneumoniae*, and 23 were excluded from the analysis due to missing TTP, to having human immunodeficiency virus infection and/or who were receiving immunosuppressant. Therefore, 278 cases were finally included in the study. Fig 1 shows the flow diagram of the study population.

### General characteristics of the study population

The median (Q_1_; Q_3_) age was 62 (46; 79) years (152 patients [55%] aged <65 yrs) and 165 (59%) of the patients were male. On admission, 142 patients (51%) were classified in the high-risk group of PSI score (IV-V). Thirty-four patients (13%) received prior antibiotic treatment and 25 (10%) had prior pneumococcal vaccination. Severe CAP, according to the ATS/IDSA criteria, was present in 82 patients (38%), pulmonary complications in 127 (47%), and extra pulmonary complication in 111 (41%) (Septic shock 30; 11% and acute renal failure 103, 38%). Eighty-seven patients (31%) were admitted to the ICU and 43 (17%) needed mechanical ventilation. Twenty-one patients (8%) died within 30 days after admission. Out of 278 isolates, 186 (67%) were available for serotyping (period 2006–2015). The most frequent serotypes in this population were 1 (n = 36, 19%), 3 (n = 20, 11%), 19A (n = 19, 10%), 7F (n = 16, 9%), and 14 (n = 11, 6%).

Minimal inhibitory concentration (MIC) testing was performed in 272 out of 278 *S*. *pneumoniae* isolates (98%). A total of 220 pneumococcal isolates (81%) were penicillin susceptible (MIC ≤ 0.06 mg/L); while 26 (10%) were intermediate (MIC 0.12–2 mg/L) and 26 (10%) were resistant (MIC > 2 mg/L). In addition, 41 pneumococcal isolates (15%) were non-susceptible to erythromycin, (MIC ≥1 mg/L).

### Time to positivity of *S*. *Pneumoniae*

**Fig 2:**
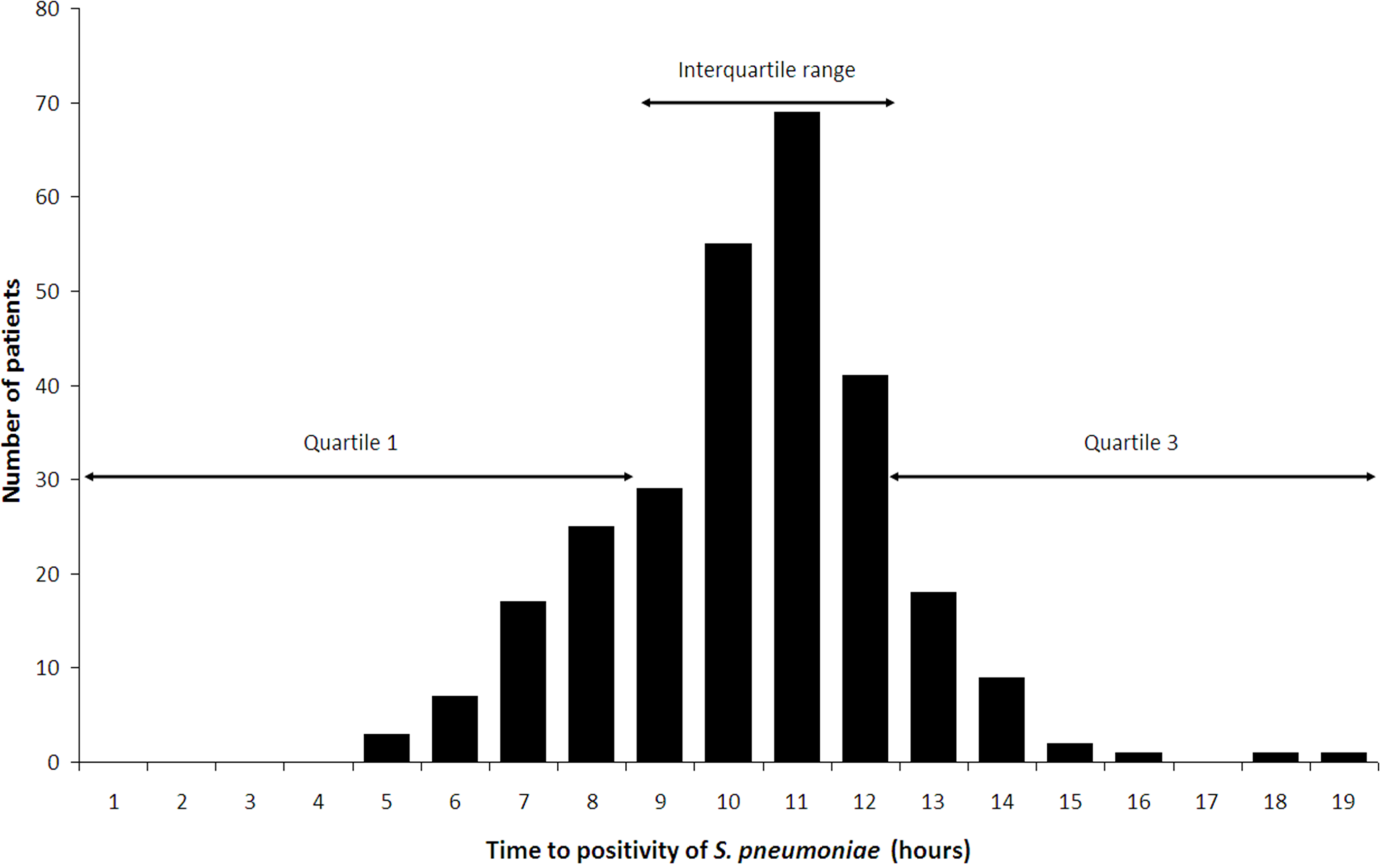
Time to positivity of *S*. *Pneumoniae* in blood culture.

**Fig 3:**
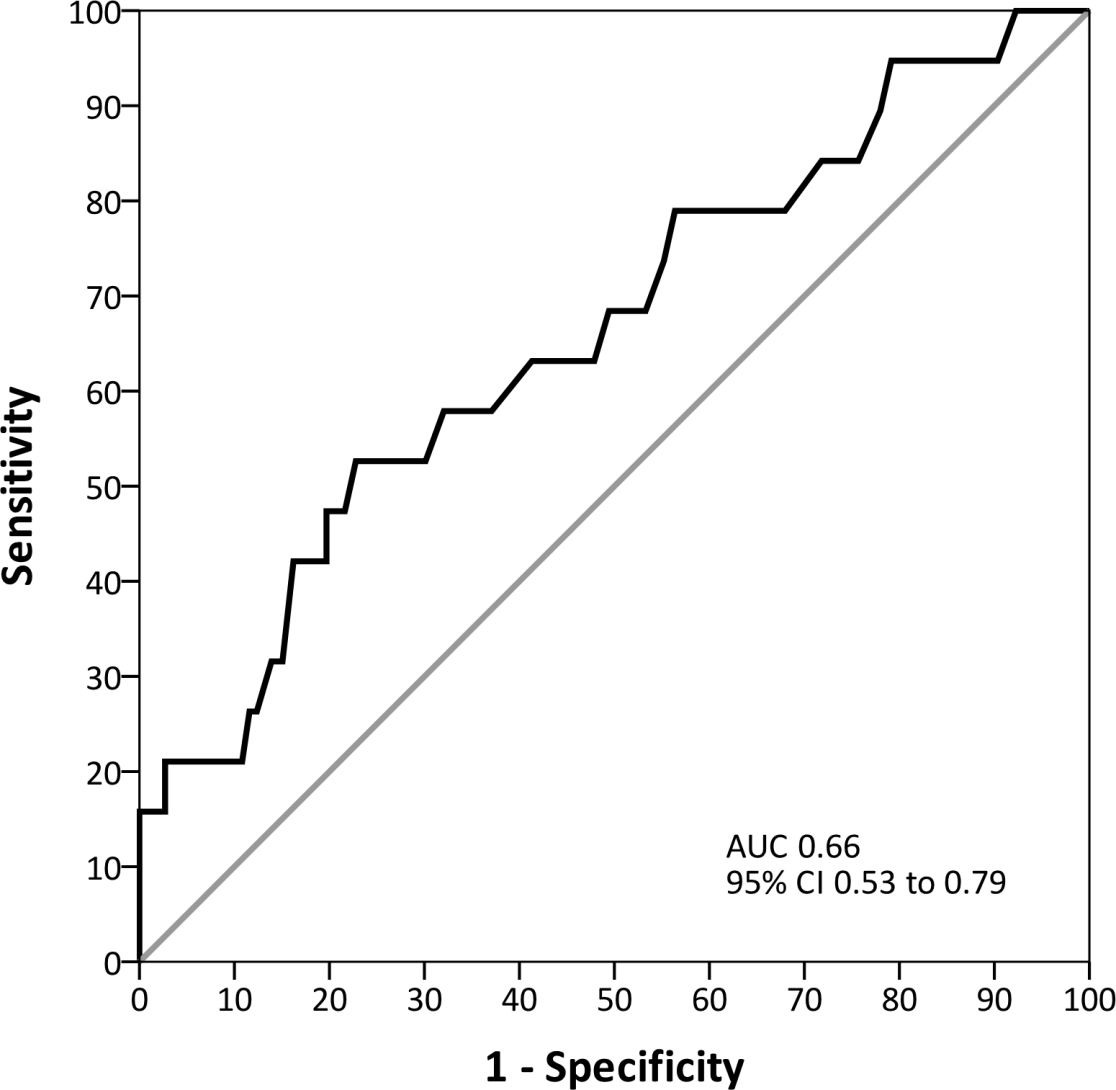
Receiver operating characteristic curve for time to positivity of *S*. *pneumoniae* to predict in-hospital mortality.

The median TTP of *S*. *pneumoniae* in blood culture among the 278 adult patients with pneumococcal CAP was 10.5 (9.2; 11.5) hours (Fig 2). Following Youden’s index methodology, we selected 9.2 h as the optimal cut-off point for TTP in relation to in-hospital mortality (Fig 3) (53% sensitivity, 77% specificity, 14% positive predictive value, 96% negative predictive value, 2.31 positive likelihood ratio, and 0.61 negative likelihood ratio). Patients were divided into two detection groups: early detection group (TTP <9.2 h: 69 patients [25%]) and late detection group (TTP ≥9.2 h: 209 patients [75%]).

Table 1 summarizes the main characteristics of the 278 patients.

**Table 1:** Baseline characteristics of study cohort.

Variables	Cohort of patients	Early detection <9.2 h	Late detection ≥9.2 h	P Value
	n = 278	n = 69	n = 209	
Age, median (IQR), years	62 (46; 79)	67 (50; 81)	60 (46; 75)	0.063
Male sex, n (%)	165 (59)	39 (57)	126 (60)	0.58
Current smoker, n (%)	93 (34)	19 (30)	74 (36)	0.35
Current alcohol abuse, n (%)	51 (19)	10 (15)	41 (20)	0.19
Previous antibiotic, n (%)	34 (13)	6 (9)	28 (14)	0.32
Influenza vaccine, n (%)	74 (31)	15 (27)	59 (32)	0.53
Pneumococcal vaccine, n (%)	25 (10)	7 (13)	18 (10)	0.53
Inhaled corticosteroid, n (%)	36 (13)	9 (13)	27 (13)	0.94
Systemic corticosteroid, n (%)	11 (4)	3 (4)	8 (4)	0.84
Comorbidity, n (%)^a^	163 (59)	37 (54)	126 (60)	0.33
Chronic respiratory disease	97 (36)	20 (30)	77 (38)	0.23
Chronic cardiovascular disease	23 (8)	7 (10)	16 (8)	0.50
Diabetes mellitus	48 (18)	13 (19)	35 (17)	0.65
Neurological disease	41 (16)	10 (15)	31 (16)	0.93
Chronic renal disease	18 (7)	4 (6)	14 (7)	0.79
Chronic liver disease	22 (8)	3 (4)	19 (2)	0.20
Clinical presentation				
Days of symptoms, median (IQR)	4 (3; 7)	4 (3; 7)	4 (2; 6)	**0.030**
Dyspnoea	183 (67)	49 (73)	134 (76)	0.25
Pleural pain	154 (57)	37 (58)	117 (57)	0.91
Fever	238 (87)	53 (79)	185 (89)	**0.031**
Laboratory findings, n (%)				
Creatinine ≥1.5 mg/dL	97 (35)	26 (38)	71 (35)	0.62
C-reactive protein ≥15 mg/dL	210 (80)	62 (91)	148 (76)	**0.008**
White blood cell count ≥10 × 10^9^/L	193 (70)	36 (52)	157 (76)	**0.001**
Lymphocytes count 10^9^/L, median (IQR)	750 (415; 1,188)	486 (244; 1,224)	792 (480; 1,188)	**0.016**
PaO_2_/FIO_2_ ratio <250	73 (39)	25 (54)	48 (34)	**0.013**
SOFA score, median (IQR)	3 (1; 4)	3 (2; 4)	2 (1; 4)	0.093
PSI score, median (IQR)	92 (66; 118)	101 (76; 124.5)	87 (65; 115)	**0.031**
PSI risk class, n (%)^b^				**0.031**
I-III	136 (49)	26 (38)	110 (53)	
IV-V	142 (51)	43 (62)	99 (47)	
CURB-65, n (%)				0.70
0–2	218 (83)	55 (81)	163 (83)	
3–5	46 (17)	13 (19)	33 (17)	
Severe CAP, n (%)	82 (38)	29 (52)	53 (33)	**0.011**
Pulmonary complications, n (%)^c^	127 (47)	40 (60)	87 (42)	**0.014**
Pleural efusion	50 (18)	18 (27)	32 (16)	**0.039**
Multilobar infiltration	93 (34)	29 (42)	64 (31)	0.082
ARDS	18 (7)	6 (9)	12 (6)	0.37
Extra-pulmonary complications, n (%)^d^	111 (41)	29 (43)	82 (40)	0.61
Acute renal failure	103 (38)	28 (41)	75 (37)	0.49
Septic shock	30 (11)	9 (13)	21 (10)	0.45
Empiric antibiotic therapy, n (%)				
ß-lactam plus fluroroquinolone	109 (39)	37 (54)	72 (35)	**0.007**
ß-lactam plus macrolide	90 (33)	19 (28)	71 (34)	0.34
Fluoroquinolone monotherapy	44 (20)	7 (10)	37 (18)	0.18
Appropriate empiric treatment, n (%)	190 (93)	54 (98)	136 (91)	0.083
Strain Penicillin resistant, n (%)	26 (10)	8 (12)	18 (9)	0.73
Strain Erithromycin resistant, n (%)	41 (15)	13 (19)	28 (14)	0.28

Abbreviations: ARDS = acute respiratory distress syndrome; CAP = community acquired pneumonia; CURB-65 = Consciousness, Urea, Respiratory rate, Blood pressure, 65 years old; IQR = interquartile range; PaO_2_/FIO_2_ = arterial oxygen tension to inspired oxygen fraction ratio; PSI = pneumonia severity index; SOFA = sequential organ failure assessment. Percentages calculated on non-missing data.

^a^ Could have more than 1 comorbid condition.

^b^ Stratified according to 30-day risk mortality for community-acquired pneumonia: risk classes I-III (≤90 points) have low mortality (range, 0%-10%) and risk classes IV-V (>90 points) have the highest mortality (range, 10%-35%).

^c^ Could have more than 1 pulmonary complication.

^d^ Could have more than 1 extra-pulmonary complication.

### Comparison of characteristics of early and late detection pneumonia

Baseline characteristics comparing cases on early detection group and late detection group appear in Table 1.

We found non-significant differences in the use of previous antibiotics and pneumococcal vaccine between groups. Compared with the late group, cases in the early detection group presented fever at admission less frequently, more days of symptoms, low white blood cell count, low lymphocyte count, higher serum levels of C-reactive protein, and worse oxygenation at admission. Also, the early detection group presented a higher median PSI score, more severe CAP according to the IDSA/ATS definition, and more pulmonary complications.

### Outcomes

The early detection group had a longer length of hospital stay, higher rate of in-hospital mortality, higher rate of 30-day mortality, and higher rate of invasive mechanical ventilation compared to the longer detection group (Table 2).

**Table 2:** Clinical outcomes.

Variables	Cohort of patients	Early detection <9.2 h	Late detection ≥9.2 h	P Value
	n = 278	n = 69	n = 209	
Length of hospital stay (days), median (IQR)	9 (5; 14)	12 (8; 18)	8 (5; 12)	**<0.001**
Length of hospital stay, ≥9 days, n (%)	139 (52)	47 (71)	92 (46)	**<0.001**
In-hospital mortality, n (%)	19 (7)	10 (15)	9 (4)	**0.010**
30-day mortality, n (%)	21 (8)	10 (15)	11 (5)	**0.018**
ICU admission, n (%)	87 (31)	27 (39)	60 (29)	0.60
Length of ICU stay (days), mean (IQR)	5 (3; 9)	5.5 (4; 21.5)	5 (3; 7)	0.16
ICU mortality, n (%)^a^	8 (9)	4 (15)	4 (7)	0.24
Mechanical ventilation, n (%)				**0.018**
Not ventilated	216 (83)	44 (70)	172 (88)	**0.029**
Non-invasive	21 (8)	8 (13)	13 (7)	0.82
Invasive	22 (8)	11 (18)	11 (6)	**0.007**

Abbreviations: ICU = intensive care unit; IQR = interquartile range. Percentages calculated on non-missing data.

^a^ 87 patients in the overall cohort, 27 patients in the early detection group and 60 patients in the late detection group were used to calculate the percentages.

Of the 21 patients who died at 30 days, approximately half were in the early detection group (10 of 21 cases [48%])

We found no difference between groups in ICU admission, length of ICU stay or ICU mortality.

**Fig 4:**
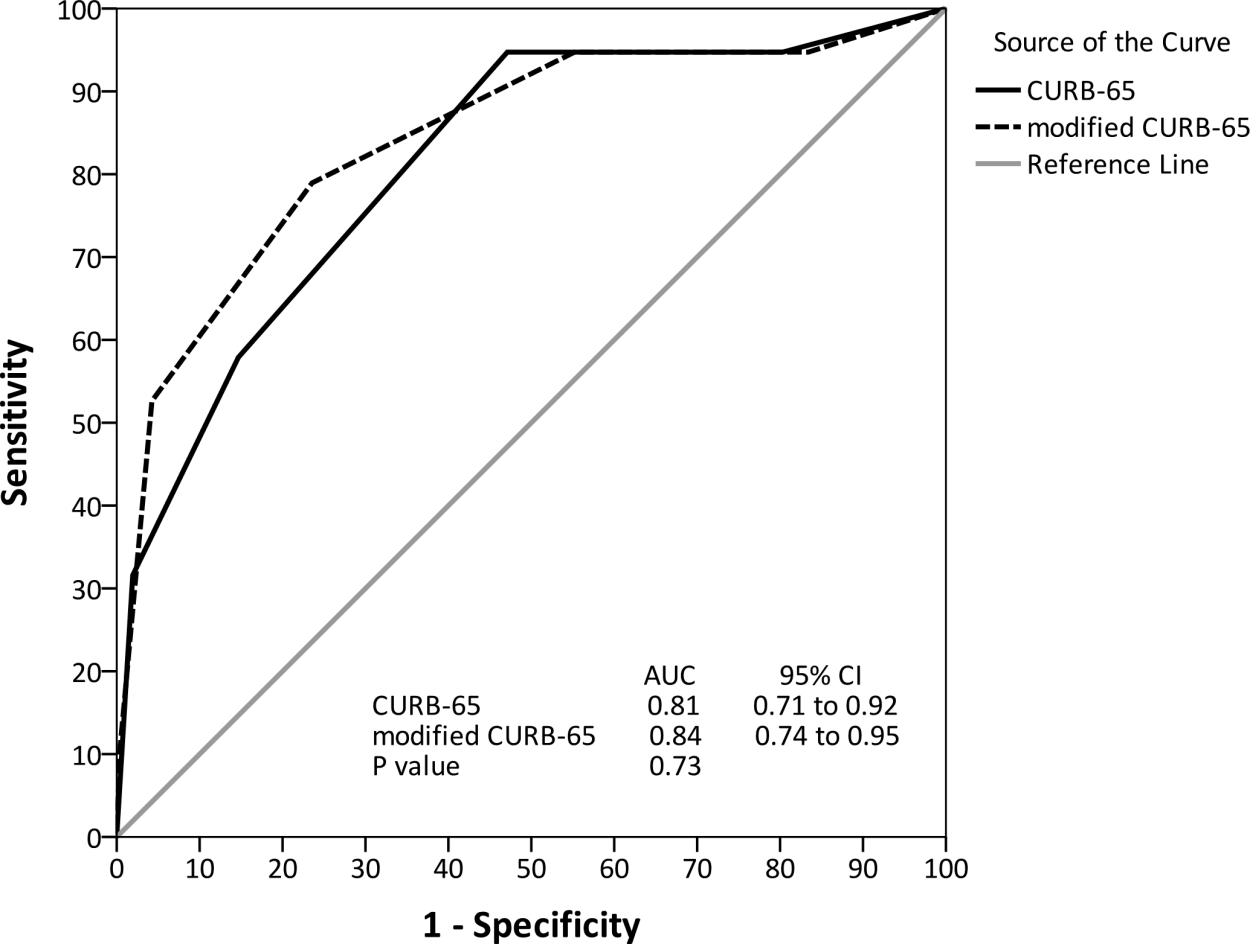
Receiver operating characteristic curve for CURB-65 and modified CURB-65 to predict in-hospital mortality.

**Fig 5:**
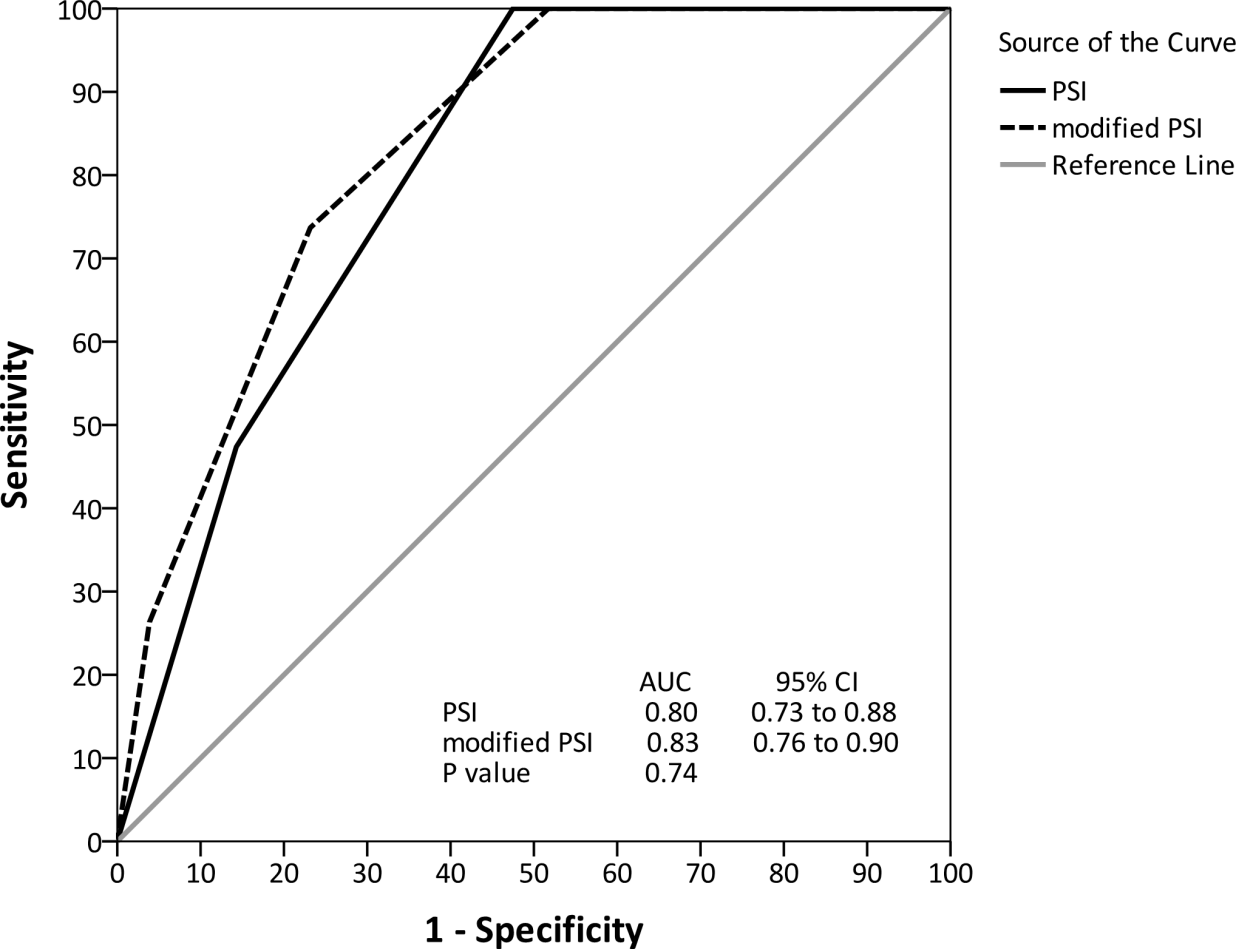
Receiver operating characteristic curve for PSI and modified PSI to predict in-hospital mortality.

We added early detection (TTP <9.2 h) to the CURB-65 and PSI scores to predict mortality, and the effectiveness of the new scores was compared. The addition of early TTP improved the area under the ROC curves of the CURB-65 (0.84, 95% CI 0.74 to 0.95) and PSI (0.83, 95% CI 0.76 to 0.90) but not reaching significance (Figs 4 and 5).

### Predictors of length of hospital stay

The simple linear regression analysis revealed several variables significantly associated with length of hospital stay (Table 3). The variables PCR ≥15 mg/dl, PSI IV-V, ARDS and early detection (TTP <9.2 h) were those independently associated with length of hospital stay in the multiple analysis.

**Table 3:** Significant simple and multiple linear regression analyses to predict length of hospital stay.

Variable	Simple			Multiple^a^^b^		
	β	95% CI	P Value	β	95% CI	P Value
Chronic respiratory disease	2.74	-0.45 to 5.93	0.092	-	-	-
C-reactive protein ≥15 mg/dL	1.68	0.92 to 3.06	0.090	-	-	-
PSI risk class IV-V	4.67	1.62 to 7.72	0.003	3.97	1.14 to 6.81	0.006
Pleural effusion	3.74	-0.21 to 7.69	0.064	-	-	-
ARDS	16.42	10.7 to 22.1	<0.001	15.58	10.1 to 21.0	<0.001
Acute renal failure	4.18	1.02 to 7.35	0.010	-	-	-
Septic shock	5.96	1.03 to 10.9	0.018	-	-	-
Mechanical ventilation	8.40	5.98 to 10.8	<0.001	-	-	-
Early detection (time to positivity <9.2 h)	6.74	3.24 to 10.24	<0.001	5.20	1.81 to 8.52	0.002

Abbreviations: β = unstandardized beta coefficient; ARDS = acute respiratory distress syndrome; CI = confidence interval; OR = odds ratio; PSI = pneumonia severity index.

Data are shown as estimated βs (95% CIs) of the explanatory variables in the model. Regression coefficients represent the mean change in the response variable for one unit of change in the predictor variable while holding other predictors in the model constant.

The P value is based on the null hypothesis that all βs relating to an explanatory variable equal zero (no effect).

^a^ Adjusted R^2^ coefficient of determination = 0.18.

^b^ Patients’ predicted length of hospital stay is equal to 4.49 + 3.97 (PSI) + 15.58 (ARDS) + 5.20 (time to positivity <9.2 h) days. Patients’ days of length of hospital stay increased 3.97 in case of PSI risk class IV-V, increased 15.58 in case of ARDS and increased 5.20 if time to positivity <9.2 h.

Internal validation of the linear regression model was conducted using bootstrapping with 1,000 samples. The three variables included in the model demonstrated robust results, with small 95% CIs around the original coefficients.

### Predictors of in-hospital mortality

The univariate logistic regression analysis revealed several variables significantly associated with in-hospital mortality (Table 4). Among these variables, age ≥65 years, acute renal failure, septic shock, ARDS, and early detection (TTP <9.2 h) were the variables independently associated with in-hospital mortality in the multivariate analysis.

**Table 4:** Significant univariate and multivariate logistic regression analyses to predict in-hospital mortality.

Variable	Univariate			Multivariate^a^^b^		
	OR	95% CI	P Value	OR	95% CI	P Value
Age ≥65 years	3.68	1.29 to 10.51	0.015	8.33	1.80 to 38.6	0.007
Alcohol consumption^b^	-	-	0.080	-	-	-
No alcohol consumption	1	-	-	-	-	-
Former alcohol consumption	1.51	0.46 to 4.95	0.50	-	-	-
Current alcohol consumption	4.17	1.20 to 14.5	0.025	-	-	-
Neurological disease	3.50	1.29 to 9.47	0.014	-	-	-
Acute renal failure	7.29	2.35 to 22.6	0.001	4.42	1.23 to 15.97	0.023
ARDS	16.40	5.50 to 48.9	<0.001	29.98	5.52 to 162.9	0.001
Septic shock	9.70	3.56 to 26.3	<0.001	5.48	1.40 to 21.4	0.014
Mechanical ventilation^d^			<0.001			-
Not ventilated	1	-	-	1	-	-
Non-invasive	7.63	2.03 to 28.6	0.003	-	-	-
Invasive	17.30	5.53 to 54.1	<0.001	-	-	-
Early detection (time to positivity <9.2 h)	3.77	1.47 to 9.70	0.006	5.35	1.55 to 18.53	0.008

Abbreviations: ARDS = acute respiratory distress syndrome; CI = confidence interval; ICU = intensive care unit; OR = odds ratio; PSI = pneumonia severity index.

Data are shown as estimated ORs (95% CIs) of the explanatory variables in the in-hospital mortality group. The OR is defined as the probability of membership of the group in-hospital mortality divided by the probability of membership of the non-in-hospital mortality group.

The P value is based on the null hypothesis that all ORs relating to an explanatory variable equal unity (no effect).

^a^ Hosmer-Lemeshow goodness-of-fit test, p = 0.18.

^b^ Predictors from the model can be used to calculate the probability of in-hospital mortality by the following formula: Exp (β)/(1+Exp(β)), where β = -6.42 + 2.12 (in case of age ≥65 years) + 3.40 (in case of ARDS) + 1.49 (in case of acute renal failure) + 1.70 (in case of septic shock) + 1.68 (if time to positivity <9.2 h).

^c^ The p-value corresponds to differences between the three groups (no alcohol consumption, former alcohol consumption or current alcohol consumption).

^d^ The p-value corresponds to differences between the three groups (not ventilated, non-invasive or invasive).

The area under the ROC curve was 0.91 (95% CI 0.85 to 0.98) for the model predictive of in-hospital mortality. Internal validation of the logistic regression model using bootstrapping with 1,000 samples demonstrated robust results for the five variables included in the model, with small 95% CIs around the original coefficients.

### Predictors of 30-day mortality

The univariate logistic regression analysis revealed several variables significantly associated with 30-day mortality (Table 5). The variables, PSI IV-V, ARDS, acute renal failure, and septic shock were those independently associated with 30-day mortality. There was a trend for early detection (TTP <9.2 h) to be a contributing factor for 30-day mortality.

**Table 5:** Significant univariate and multivariate logistic regression analyses to predict 30-day mortality.

Variable	Univariate			Multivariate^a^^b^		
	OR	95% CI	P Value	OR	95% CI	P Value
Age ≥65 years	3.29	1.24 to 8.75	0.017	-	-	-
Chronic cardiovascular disease	2.95	0.90 to 9.64	0.074	-	-	-
Neurological disease	3.78	1.46 to 9.75	0.006	-	-	-
PSI risk class IV-V	22.13	2.93 to 167.3	0.003	19.93	2.47 to 161.1	0.005
Acute renal failure	6.25	2.22 to 17.6	0.001	-	-	-
ARDS	13.76	4.73 to 44.0	<0.001	11.13	2.68 to 46.2	0.001
Septic shock	8.01	3.04 to 21.10	<0.001	3.95	1.17 to 13.56	0.027
Mechanical ventilation^c^			<0.001			-
Not ventilated	1	-	-	1	-	-
Non-invasive	5.88	1.64 to 21.0	0.007	-	-	-
Invasive	13.33	4.50 to 39.5	<0.001	-	-	-
Early detection (time to positivity <9.2 h)	3.05	1.23 to 7.54	0.016	2.47	0.85 to 7.21	0.097

Abbreviations: ARDS = acute respiratory distress syndrome; CI = confidence interval; ICU = intensive care unit; OR = odds ratio; PSI = pneumonia severity index.

Data are shown as estimated ORs (95% CIs) of the explanatory variables in the 30-day mortality group. The OR is defined as the probability of membership of the group 30-day mortality divided by the probability of membership of the non-30-day mortality group.

The P value is based on the null hypothesis that all ORs relating to an explanatory variable equal unity (no effect).

^a^ Hosmer-Lemeshow goodness-of-fit test, p = 0.51.

^b^ Predictors from the model can be used to calculate the probability of 30-day mortality by the following formula: Exp(β)/(1+Exp(β)), where β = -5.81 + 2.99 (in case of PSI risk class IV-V) + 2.41 (in case of ARDS) + 1.37 (in case of septic shock) + 0.91 (if time to positivity <9.2 h).

^c^ The p-value corresponds to differences between the three groups (not ventilated, non-invasive or invasive).

The area under the ROC curve was 0.88 (95% CI 0.79 to 0.96) for the model predictive of 30-day mortality. Internal validation of the logistic regression model using bootstrapping with 1,000 samples demonstrated robust results for three of four variables included in the model, with small 95% CIs around the original coefficients, while the PSI appeared to be less reliable, with a wider 95% CI around the original coefficient.

### Predictors of the need for non-invasive or invasive mechanical ventilation

The following risk factors showed significant associations with mechanical ventilation groups in individual multinomial logistic regression and were thus used for the initial multivariate model: alcohol consumption, chronic respiratory disease, lymphocytes, PSI risk class, ARDS, and septic shock and early detection time. Results of the multivariate model are displayed in Table 6. For non-invasive mechanical ventilation, the model shows the OR to be significantly increased if they have prior alcohol consumption, PSI risk class IV-V, and septic shock. The OR for invasive mechanical ventilation, however, was strongly increased with septic shock and early detection (TTP <9.2 h). The area under the ROC curve was 0.75 (95% CI 0.65 to 0.84) for the model predictive of non-invasive mechanical ventilation, and 0.81 (95% CI 0.70 to 0.91) for the model predictive of invasive mechanical ventilation.

**Table 6:** Multivariate multinomial logistic regression analyses to predict non-invasive or invasive mechanical ventilation relative to non-ventilated.

Variable	Non-invasive mechanical ventilation			Invasive mechanical ventilation		
	OR	95% CI	P Value	OR	95% CI	P Value
Alcohol consumption						
No alcohol consumption	1	-	-	1	-	-
Current alcohol consumption	2.45	0.75 to 7.94	0.14	3.14	0.99 to 9.97	0.053
Former alcohol consumption	4.84	1.24 to 18.95	0.024	3.24	0.64 to 16.49	0.16
PSI IV-V	4.83	1.52 to 15.40	0.008	2.09	0.73 to 6.02	0.17
Septic shock	3.89	1.15 to 13.17	0.029	13.99	4.75 to 41.20	0.001
Early detection (time to positivity <9.2 h)	2.25	0.83 to 6.11	0.11	4.60	1.63 to 13.03	0.004

Abbreviations: ARDS = acute respiratory distress syndrome; CI = confidence interval; OR = odds ratio; PSI = pneumonia severity index.

Data are shown as estimated ORs (95% CIs) of the explanatory variables in the non-invasive mechanical ventilation and invasive mechanical ventilation groups. The OR is defined as the probability of membership of the groups non-invasive or invasive divided by the probability of membership of the not ventilated group.

The P value is based on the null hypothesis that all ORs relating to an explanatory variable equal unity (no effect).

Model characteristics: likelihood ratio χ^2^ test, p = 0.17; R^2^ coefficients = 0.19 (Cox and Snell), 0.29 (Nagelkerke).

Predictors from the model can be used to calculate the probability of non-invasive mechanical ventilation or invasive mechanical ventilation by the following formulas: Exp(β_1_)/(1+Exp(β_1_)+Exp(β_2_)) and Exp(β_2_)/(1+Exp(β_1_)+Exp(β_2_)), respectively, where β_1_ = -4.13 + 1.58 (in case of former alcohol consumption) + 0.98 (in case of current alcohol consumption) + 1.58 (in case of PSI risk class IV-V) + 1.36 (in case of septic shock) + 0.81 (if time to positivity <9.2 h) and β_2_ = -4.24 + 1.18 (in case of former alcohol consumption) + 1.14 (in case of current alcohol consumption) + 0.74 (in case of PSI risk class IV-V) + 2.64 (in case of septic shock) + 1.53 (if time to positivity <9.2 h).

Internal validation of the multinomial logistic regression model using bootstrapping with 1,000 samples demonstrated robust results for the four variables included in the model, with small 95% CIs around the original coefficients.

### Relationship between time to positivity and pneumococcal serotypes

In order to analyze the association between pneumococcal serotypes and time to positivity, pneumococcal serotypes were divided into three groups according to invasiveness: low (serotypes 3, 6A, 6B, 19A, 19F and 23F), intermediate (4, 9N, 9V, 14 and 18C) or high (1, 5 and 7F). We did not find any association between TTP and serotype (early detection group (TTP <9.2 h): low 30%, intermediate 19%, high 51% vs. late detection group (TTP ≥9.2 h): low 35%, intermediate 22%, high 43%, p = 0.67).

There were no differences in the rate of resistance to penicillin (12% vs. 9%, p = 0.73) nor macrolides (19% vs. 14%, p = 0.28) between groups.

## Discussion

A TTP of blood culture shorter than 9.2h in patients with bacteremic pneumococcal pneumonia is independently associated with a more severe disease characterized by a higher C-reactive protein level, a worst oxygenation, and more pulmonary complications which explain the more frequent need of invasive mechanical ventilation in the early detection group. However, we did not find an association between TTP <9.2 h and septic shock, which could be due to the lower number of septic shock patients. In line with this, a TTP <9.2 h was independently associated with the risk of mechanical ventilation, longer length of hospital stay, in-hospital mortality and there was a trend towards higher 30-day mortality. These findings concord with existing data that describe the strong association between the bacterial load and clinical outcomes in invasive pneumococcal diseases [18,23]. These studies were performed with the polymerase chain reaction and showed a higher sensitivity in comparison to blood cultures, although this method cannot measure the viability of the bacteria. Also, our results are in accordance with the results of study by Werno et al. that reported the association of higher pneumococcal load with increased disease severity in adults with CAP [6].

The concept of early TTP of blood cultures is directly related with a higher bacterial concentration in blood. Interestingly, the first finding of our study is related to the pathogenesis of pneumococcal infection. Patients with blood cultures with rapid growth of pneumococcus could be due to a dysregulation in the host innate immune response, shown by the low number of circulating leukocytes and a high inflammatory response measured by high levels of C-reactive protein [23–27].

These results emphasize that the key to improve the prognosis of patients with bacteremic pneumococcal pneumonia is to improve the host immune response against pneumococcus, and to learn to modulate the inflammatory response of the host. A higher bacterial load could lead to a higher inflammatory response when a β-lactam antibiotic is used [5]; this effect was observed in meningitis and sepsis [28] and was proposed as the key for benefits of corticosteroids, macrolides or fluoroquinoles in adjuvant treatment of pneumonia [29–33].

TTP was better studied in child patients with bacteremic disease by several pathogens such as *S*. *pneumoniae*, *N*. *meningitidis and H*. *influenzae*. A higher bacterial load in blood correlates with severity of the disease, also the bacterial load correlates with TTP in catheter related sepsis [34]. In paediatric patients there was no relation among TTP and severity although a positivity correlation was described in adults with pneumococcal invasive disease [8]. A recent small study about the relation of fluorescence rate using blood culture techniques and mortality in patients with invasive pneumococcal pneumonia found a direct association of FR and mortality. However, the authors did not find that TTP correlated with clinical outcomes [35].

Interestingly, no statistically significant differences were observed in the use of previous antibiotics and pneumococcal vaccine in our cohort of patients between groups. Also, there were no differences in resistance to penicillin or macrolides.

We found that early TTP was an independent risk factor for in-hospital mortality; however, only a trend for 30-day mortality was observed. This could be due to an under power analysis.

The prediction models we have presented are the first step in establishing more universal models; to move forward, our prediction models will need to undergo external validations with larger patient cohorts from multiple centers. We were able to apply internal validation techniques to understand how likely these models will be replicable in future studies and at other centers. Bootstrapping techniques were applied and demonstrated that the coefficients obtained from these prediction models were quite robust. PSI was the one factor for 30-day mortality model that the bootstrap results indicated might have limited repeatability in future work. Removal of the PSI from the model did not change which factors were significant predictors of 30-day mortality. However, because of the clinical importance surrounding the PSI, this variable was kept as a factor in this model despite some statistical limitations. In the real world clinical setting where this prediction model could be used, the PSI is an important clinical characteristic that can play a substantial role in decision making.

To the best of our knowledge, with the exception of one study [8] evaluating patients with invasive pneumococcal disease, this is the first study addressing the issue of early TTP specifically in pneumococcal pneumonia in a large adult population.

TTP is an easy to obtain parameter available in all Microbiology laboratories that appears to provide useful prognostic information, and should be reported routinely in order to help clinicians to identify patients at risk of worse outcome that could benefit from more aggressive early management.

Several limitations have to be addressed. First, because the data were collected from a single academic teaching hospital in Spain the results might not be able to be extrapolated to other patients admitted to other types of hospitals in other countries. Second, we could only analyze 278 patients and this sample size may result in a large type II error. Our sample size, however, is large since this is the only study regarding this issue. Our results support the direct relationship of early TTP with severe presentation and worse outcomes in patients with diagnosis of pneumococcal CAP.

### Conclusion

In summary, we found evidence that in those patients with pneumococcal pneumonia that had early TTP, they were more severely ill at presentation and had worse outcomes.

## Supporting information

S1 Database (SAV)
